# An Analysis of the Novel Fluorocycline TP-6076 Bound to Both the Ribosome and Multidrug Efflux Pump AdeJ from Acinetobacter baumannii

**DOI:** 10.1128/mbio.03732-21

**Published:** 2022-02-01

**Authors:** Christopher E. Morgan, Zhemin Zhang, Robert A. Bonomo, Edward W. Yu

**Affiliations:** a Department of Pharmacology, Case Western Reserve University School of Medicine, Cleveland, Ohio, USA; b Louis Stokes Cleveland Department of Veterans Affairs Medical Center, Cleveland, Ohio, USA

**Keywords:** *Acinetobacter baumannii*, AdeJ, TP-6076, multidrug efflux pump, multidrug resistance, ribosomes

## Abstract

Antibiotic resistance among bacterial pathogens continues to pose a serious global health threat. Multidrug-resistant (MDR) strains of the Gram-negative organism Acinetobacter baumannii utilize a number of resistance determinants to evade current antibiotics. One of the major resistance mechanisms employed by these pathogens is the use of multidrug efflux pumps. These pumps extrude xenobiotics directly out of bacterial cells, resulting in treatment failures when common antibiotics are administered. Here, the structure of the novel tetracycline antibiotic TP-6076, bound to both the Acinetobacter drug efflux pump AdeJ and the ribosome from Acinetobacter baumannii, using single-particle cryo-electron microscopy (cryo-EM), is elucidated. In this work, the structure of the AdeJ–TP-6076 complex is solved, and we show that AdeJ utilizes a network of hydrophobic interactions to recognize this fluorocycline. Concomitant with this, we elucidate three structures of TP-6076 bound to the A. baumannii ribosome and determine that its binding is stabilized largely by electrostatic interactions. We then compare the differences in binding modes between TP-6076 and the related tetracycline antibiotic eravacycline in both targets. These differences suggest that modifications to the tetracycline core may be able to alter AdeJ binding while maintaining interactions with the ribosome. Together, this work highlights how different mechanisms are used to stabilize the binding of tetracycline-based compounds to unique bacterial targets and provides guidance for the future clinical development of tetracycline antibiotics.

## INTRODUCTION

The tetracycline class of antibiotics is composed of powerful antimicrobials that are widely used in modern medicine (1). Tetracyclines show a broad spectrum of inhibition of Gram-negative, Gram-positive, and anaerobic bacteria, allowing their use in the treatment of a number of different types of infections (1–3). TP-6076 is a fully synthetic tetracycline class antimicrobial agent. This novel antibiotic was originally developed by TetraPhase Pharmaceutics and is classified as a fluorinated-hydrocarbon antibacterial. This compound acts as a bacterial 70S ribosome inhibitor, specifically targeting the small 30S subunit of the ribosome to halt bacterial protein expression (1). Recently, this ribosome inhibitor has completed a phase I trial. It is a powerful drug that shows enhanced antibacterial activity against troublesome Gram-negative and Gram-positive pathogens compared with previous generations of tetracycline antibiotics (4, 5).

Tetracyclines typically function by binding directly to the bacterial ribosome (6). The ribosome is a 2.3-MDa complex that is responsible for protein synthesis inside the cell (7). The complete ribosome, the 70S ribosome, is made up of the 50S subunit and the 30S subunit (7–9). The large 50S subunit, which contains two rRNAs, 23S and 5S, and 34 proteins, is responsible for binding aminoacyl tRNA, catalyzing peptidyl transfer and peptide elongation (7). The A, P, and E tRNA sites of the 70S ribosome bind aminoacylated tRNA, dock peptidyl-tRNA representing the elongating polypeptide chain, and engage/release deacylated tRNA, respectively. The small 30S subunit, which contains the 16S rRNA and 21 proteins, is responsible for the binding of mRNA, ensuring coding fidelity and initiating protein synthesis (7). The proper function of the ribosome is vital for the survival of the cell.

Because of its complex and coordinated functions, the bacterial ribosome contains many sites that can be targeted by antibacterial agents. Studies have shown that different antibiotic compounds can bind to specific regions of this complex biomacromolecular machine (10, 11). For example, aminoglycoside-based antibiotics interact with the 30S subunit to inhibit proofreading functionality, while macrolides bind to the peptide exit tunnel of the 50S subunit to inhibit polypeptide chain elongation. For tetracycline-based compounds, ribosome inhibition is achieved by direct interaction with the 30S subunit to block the binding of tRNA to the A site. This inhibits the elongation of the polypeptide chain, and translation halts (11).

While currently effective, the potency of many antibiotics is expected to diminish with time, likely due to both misuse and bacterial adaptation that leads to enhanced antimicrobial resistance (AMR) (12). Multidrug-resistant (MDR) bacterial pathogens largely utilize several different mechanisms to evade tetracycline antibiotics. The two most common are the overexpression of multidrug efflux pumps and the expression of ribosomal protection proteins (2, 6, 13).

Gram-negative MDR bacterial pathogens, including Acinetobacter baumannii, commonly utilize the upregulation of the resistance-nodulation-cell division (RND) superfamily of transporters to mediate resistance to multiple antimicrobial agents (14–18). Typically, an RND multidrug efflux system forms a large tripartite protein complex spanning the entire cell envelope to expel antibiotics and other toxic compounds from bacterial cells. These efflux pumps are potent defense systems due to their ability to confer simultaneous resistance to multiple classes of antibiotics.

A. baumannii is one of the most dangerous MDR bacterial strains to threaten the lives of vulnerable patients, as this pathogen exhibits a high level of drug resistance to a broad range of antimicrobial agents. Emerging carbapenem-resistant A. baumannii is now listed in the highest-urgency AMR threat category by the Centers for Disease Control and Prevention (CDC). The World Health Organization (WHO) also classifies carbapenem-resistant A. baumannii as a “first-priority pathogen” for the research and development of new antibiotics.

The principal RND transporter that endows tetracycline resistance in A. baumannii is AdeIJK (19–24). AdeIJK is a tripartite multidrug efflux system that spans the entire bacterial cell envelope (25, 26). The inner membrane component of the system, AdeJ, is responsible for drug recognition and powering substrate extrusion by utilizing proton motive force (PMF) as its energy source (25). Compounds are transported from AdeJ through AdeI, the periplasmic membrane fusion protein, to AdeK, the outer membrane channel, potentiating the active removal of substrates from the cell (25).

In order to better understand the determinants of how TP-6076 is recognized by AdeJ and the bacterial ribosome, cryo-electron microscopy (cryo-EM) was used to solve the structures of each complex. Here, we present the first structures of TP-6076 bound separately to the A. baumannii ribosome and the AdeJ pump. Additionally, we report the first structure of the AdeJ–TP-6076 complex to a resolution of 2.91 Å and three distinct structures of the ribosome–TP-6076 complex to resolutions of between 2.38 Å and 3.05 Å. Our results show that TP-6076 binds via very different modes of interaction with AdeJ and the ribosome. Compared to the tetracycline antibiotic eravacycline (Era)-bound structures, we show that modifications to the tetracycline core in positions 4 through 8 of TP-6076 can significantly alter binding to AdeJ with little to no change in its ribosome-bound position (27). Together, our work contributes key insights into the recognition of TP-6076 by AdeJ and the ribosome and provides a guide for future generations of tetracycline development with the goal of evading extrusion while maintaining antimicrobial function.

## RESULTS

### Structure of the A. baumannii AdeJ–TP-6076 complex.

**FIG 1:**
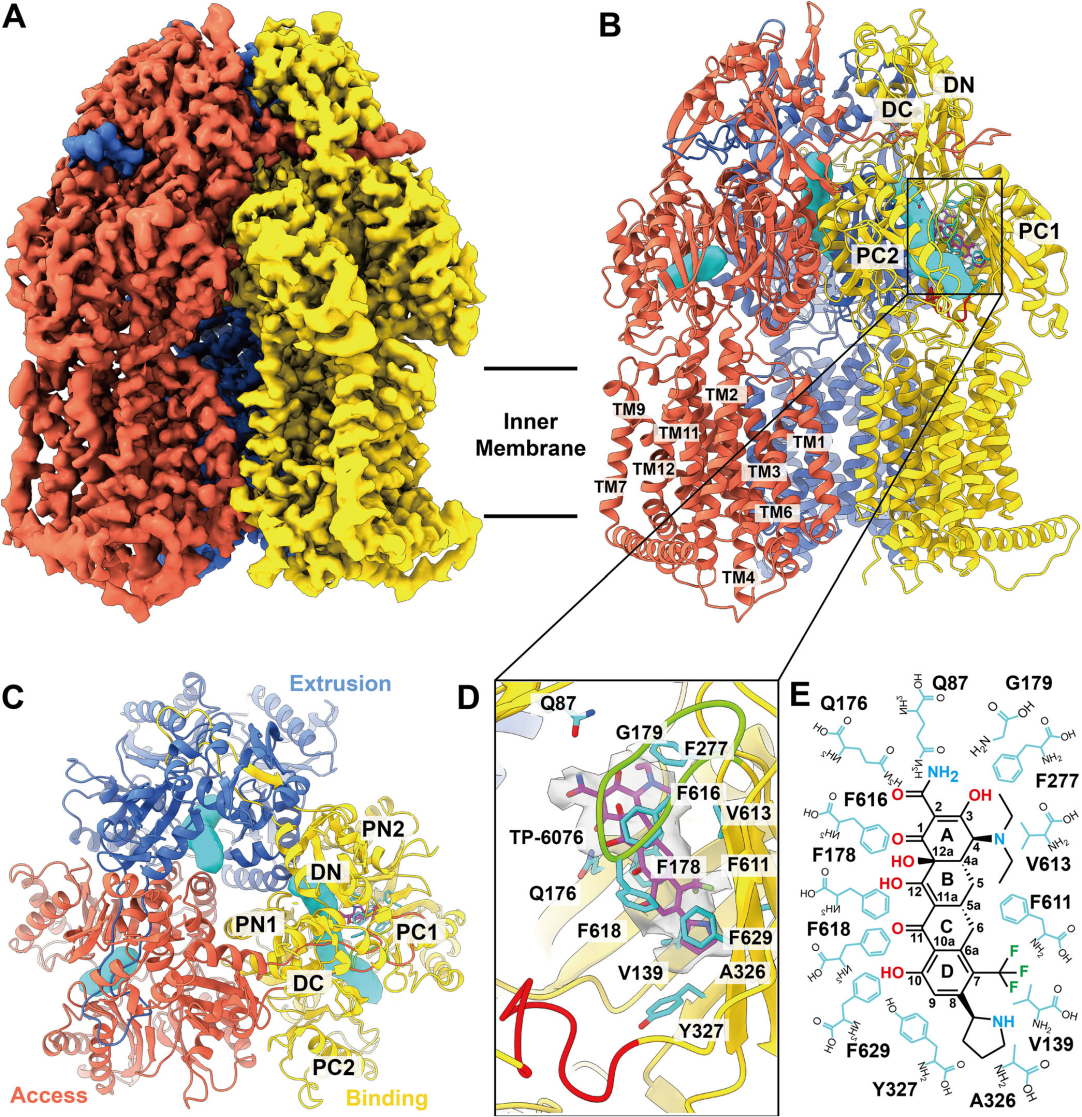
Cryo-EM structure of the A. baumannii AdeJ multidrug efflux pump bound by TP-6076. (A) Side view of the sharpened cryo-EM map of AdeJ–TP-6076. The access, binding, and extrusion state protomers are labeled in tomato, gold, and royal blue, respectively. In the figures shown throughout this paper, unless indicated otherwise, colors are assigned in the same scheme. (B) Ribbon diagram of the AdeJ–TP-6076 trimer viewed from the same direction as in panel A with the distal drug binding site displaying bound TP-6076 (magenta). Periplasmic domains are labeled in the binding state protomer. Transmembrane helices are labeled in the access protomer. Periplasmic domains are labeled in the binding state protomer (PC1 and PC2 subdomains, C-terminal periplasmic 1 and 2 subdomains; DN and DC subdomains, N-terminal and C-terminal docking subdomains). Transmembrane (TM) helices are labeled in the access protomer. Periplasmic drug tunnels are labeled as cyan surfaces. (C) Top view of the periplasmic domains of the AdeJ–TP-6076 trimer. (D) Enlarged view of the TP-6076 binding site. Residues that participate in TP-6076 binding are in cyan sticks. The density of TP-6076 is presented as a surface. The G-loop is labeled in lawn green. The F-loop is shown in red. (E) 2D diagram of the interactions between AdeJ and TP-6076. Amino residues are in cyan.

To determine how A. baumannii AdeJ recognizes the TP-6076 substrate, we utilized single-particle cryo-EM to determine its structure to high resolution. A. baumannii AdeJ was expressed in Escherichia coli, purified, and reconstituted into lipidic nanodiscs. The sample was incubated with TP-6076 to form the AdeJ–TP-6076 complex, and the structure of the complex was solved (Fig. 1; see also Fig. S1 and Table S1 in the supplemental material). Overall, at least 1,046 residues of the 1,056 residues from full-length AdeJ were built into each model.

10.1128/mbio.03732-21.1 FIG S1 Data processing. (A) Data processing workflow of apo-AdeJ and AdeJ–TP-6076. (B) Representative 2D classes of AdeJ–TP-6076. (C). Gold-standard Fourier shell correlation (GS-FSC) curve of AdeJ–TP-6076. (D and E). Side and top views of AdeJ–TP-6076 density maps. Chains in AdeJ–TP-6076 are labeled in tomato, gold, and royal blue. Download FIG S1, JPG file, 2.3 MB. Copyright © 2022 Morgan et al. 2022 Morgan et al. https://creativecommons.org/licenses/by/4.0/ This content is distributed under the terms of the Creative Commons Attribution 4.0 International license.

10.1128/mbio.03732-21.5 TABLE S1 AdeJ cryo-EM data collection and refinement statistics. Download Table S1, DOCX file, 0.02 MB. Copyright © 2022 Morgan et al. 2022 Morgan et al. https://creativecommons.org/licenses/by/4.0/ This content is distributed under the terms of the Creative Commons Attribution 4.0 International license.

AdeJ assembles as a homotrimer and adopts the typical fold of a hydrophobe-amphiphile efflux (HAE)-RND pump (26). In agreement with previous studies of AdeJ (27), each AdeJ protomer consists of a periplasmic and a transmembrane region. The transmembrane domain contains 12 transmembrane helices (TMs 1 to 12), while the periplasmic domain consists of a portal domain (PN1, PN2, PC1, and PC2) and a docking domain (DN1 and DN2) (Fig. 1B to D).

Similar to our previous studies (27), a periplasmic cleft is formed between the PC1 and PC2 subdomains (Fig. 1B and C). This cleft forms an entrance binding site for ligand interaction prior to shuttling past the flexible loop (F-loop) in the A. baumannii AdeB multidrug efflux pump (28). Presumably, the integrity of this site is important for drug recognition, where residues M666, L668, R701, R718, and T831 are coordinated to surround this entrance site. We emphasize that the corresponding residues of R718 in Escherichia coli AcrB (residue R717) and Pseudomonas aeruginosa MexB (residue R716) have previously been determined to be important for drug recognition (29, 30).

The F-loop in RND-type efflux pumps leads the drug molecule from the entrance to the proximal binding site. As indicated previously (27), the F-loop in AdeJ consists of residues 671 to 680 that connect the cleft entrance to the proximal site. This loop is likely flexible, similar to the corresponding F-loop in the AdeB pump (28).

The F-loop also creates the bottom portion of the proximal binding site, which is made up of at least 22 residues in E. coli AcrB (31). Of these 22 residues, S79, Q579, F618, E675, L676, G671, and G721 of AdeJ are conserved in E. coli AcrB (32, 33), Neisseria gonorrhoeae MtrD (34, 35), and A. baumannii AdeB (28, 36).

The gate loop (G-loop), which shuttles the ligand from the proximal to distal binding sites prior to extrusion, is formed by residues 615 to 624 in AdeJ. In AdeB, the G-loop has been shown to be flexible and occupy different conformations in apo and bound protomers (28). In addition, the conserved phenylalanine residue F612 of the AdeB G-loop (F618 in AdeJ) is actively engaged in anchoring the bound ligand at the distal site (28).

In AcrB, the distal binding site is made up of more than 20 residues (32, 33). Six of the corresponding distal binding site residues in AdeJ (F136, F178, Y327, V613, F618, and F629) are also conserved in AdeB (28, 36), AcrB (32, 33), and MtrD (34, 35). In addition, F277 from AdeJ is conserved in AdeB and AcrB, while M575 and F616 in AdeJ are also conserved in AcrB and MtrD. The hydrophobic patch, previously shown in AcrB to be vital for ligand export, is made up of F178, F277, V613, and F616 in AdeJ.

In the TP-6076-bound AdeJ trimer, each protomer occupies a distinct structural state forming an asymmetric trimer (Fig. 1A to C). These different states are vital for drug export, as seen in previous fluorescence resonance energy transfer (FRET) studies of Campylobacter jejuni CmeB (37). Using three metrics for classification (Fig. S2 and Table S2), the AdeJ–TP-6076 complex was determined to consist of binding, access, and extrusion protomers. This agrees with our previous studies of AdeJ bound to eravacycline and with previous cryo-EM studies of AdeB (27, 28).

10.1128/mbio.03732-21.2 FIG S2 AdeJ structural state assignment. (A, top) Distance between DN and DC domains (exit tunnel) in the AdeJ access state. (Bottom) The proton relay network of the AdeJ access state. DN and DC domain distances of the three apo-AdeJ protomers are measured between the C_α_ atoms of Q125 and Y759. Residues involved in the proton relay network (D407, D408, K952, N953, and T989) are shown as cyan sticks and surface density. Hydrogen bonds between K952 and D407, D408, K953, and T989 are labeled with yellow dashes. (B) AdeJ binding state. (C) AdeJ extrusion state. Download FIG S2, JPG file, 2.2 MB. Copyright © 2022 Morgan et al. 2022 Morgan et al. https://creativecommons.org/licenses/by/4.0/ This content is distributed under the terms of the Creative Commons Attribution 4.0 International license.

10.1128/mbio.03732-21.6 TABLE S2 Classification of AdeJ protomer states. Download Table S2, DOCX file, 0.01 MB. Copyright © 2022 Morgan et al. 2022 Morgan et al. https://creativecommons.org/licenses/by/4.0/ This content is distributed under the terms of the Creative Commons Attribution 4.0 International license.

The entrance site of the access protomer of AdeJ is open, whereas its extrusion site is closed (Fig. 1B and C). While a tunnel can be traced through the cleft entrance to the proximal binding pocket, a ligand is not found in the protomer.

Unlike the access protomer, the conformation of the extrusion protomer depicts that the periplasmic cleft between subdomains PC1 and PC2 is closed. Therefore, the entrance site of this protomer is also closed. An extrusion tunnel can be traced from the distal binding site out through the extrusion site, which is open in this conformational state. Presumably, this tunnel allows the ligand to be extruded from the distal binding pocket.

In the binding protomer, the entrance site between PC1 and PC2 is open (Fig. 1B and C). A binding tunnel can be traced from the entrance site through the F-loop, the proximal binding pocket, and the G-loop and into the distal binding pocket. This binding tunnel is the presumed path for the ligand to travel from the periplasmic entrance into the distal binding pocket. This agrees with previous molecular dynamics (MD) simulation studies of AdeB (28), where an ethidium molecule was found to follow this path upon binding to the distal pocket.

An extra density was observed in the binding protomer of AdeJ deep within the periplasmic cleft (Fig. 1D). This density corresponds to the bound TP-6076 antibiotic at the distal pocket of the binding protomer. The binding of TP-6076 is largely stabilized by both aromatic and hydrophobic interactions. Ten hydrophobic residues of the distal site anchor the four-ring system of the tetracycline core of the antibiotic (Fig. 1D and E). These 11 residues are V139, F178, G179, F277, A326, Y327, F611, V613, F616, F618, and F629. Particularly, F178 and F616 participate in π-π stacking interactions with rings A and C, respectively. Additionally, V139 and F611 interact with the trifluoromethyl group of ring D, while G179, V613, and F277 participate in alkyl-alkyl and π-alkyl interactions with the diethylamino group on ring A. A326, Y327, and F629 are also engaged in alkyl-alkyl and π-alkyl interactions with the pyrrolidine group of ring D. Electrostatic interactions between Q87 and Q176 and the carboxamide group on ring A of the tetracycline core further stabilize TP-6076 binding to the distal pocket of AdeJ. As Mg^2+^ is known to coordinate with tetracyclines in the ribosome, it is somewhat surprising that no Mg^2+^ ions were found to bind the TP-6076 drug in the AdeJ–TP-6076 structure. However, this observation agrees with the X-ray structure of AcrB in complex with minocycline and the cryo-EM structure of AdeB in complex with eravacycline (27, 38).

RND pumps utilize proton motive force (PMF) to extrude ligands from the periplasmic domain (27, 28, 35). In AdeJ, this force is most likely generated via the proton relay network established by the conserved residues D407, D408, K952, N953, and T989 in the transmembrane domain of each protomer. As in the previous studies of AdeB, AdeJ, and MtrD, our structural data indicate concerted motions between the rearrangement of hydrogen bonds in this proton relay network, where it couples with conformational changes between the resting, access, binding, and extrusion states of the AdeJ multidrug efflux pump (Fig. S2).

### Structural analysis of the A. baumannii 70S ribosome.

As tetracyclines target the bacterial 70S ribosome to inhibit protein synthesis, we solved the structure of TP-6076 bound to the A. baumannii ribosome to better understand the mode of the tetracycline-scaffold-ribosome interaction. The A. baumannii 70S ribosome was incubated with TP-6076 for 1 h, cryo-EM grids were prepared, and cryo-EM data were collected for this ribosome–TP-6076 complex. Using extensive classification, we were able to solve three separate cryo-EM structures of the A. baumannii 70S ribosome bound to TP-6076. These three 70S–TP-6076 structures are designated P-site tRNA, E-site tRNA, and empty 70S ribosome structures (Fig. S3).

10.1128/mbio.03732-21.3 FIG S3 Cryo-EM workflow of ribosome–TP-6076 processing. (A) Initial processing of 2,922 micrographs resulted in initial maps of assembled A. baumannii 70S ribosomes along with individual 50S and 30S subunits. (B) A modified build-and-retrieve protocol was used to separate the data set and increase particle counts. (C) Only 70S ribosomal particles were taken for further processing. (D) 3D variability analysis was used to separate 70S particles based on the tRNA population. (E and F) Homogeneous refinement followed by local refinement, CTF refinement, and density modification resulted in final maps. Download FIG S3, JPG file, 2.1 MB. Copyright © 2022 Morgan et al. 2022 Morgan et al. https://creativecommons.org/licenses/by/4.0/ This content is distributed under the terms of the Creative Commons Attribution 4.0 International license.

**FIG 2:**
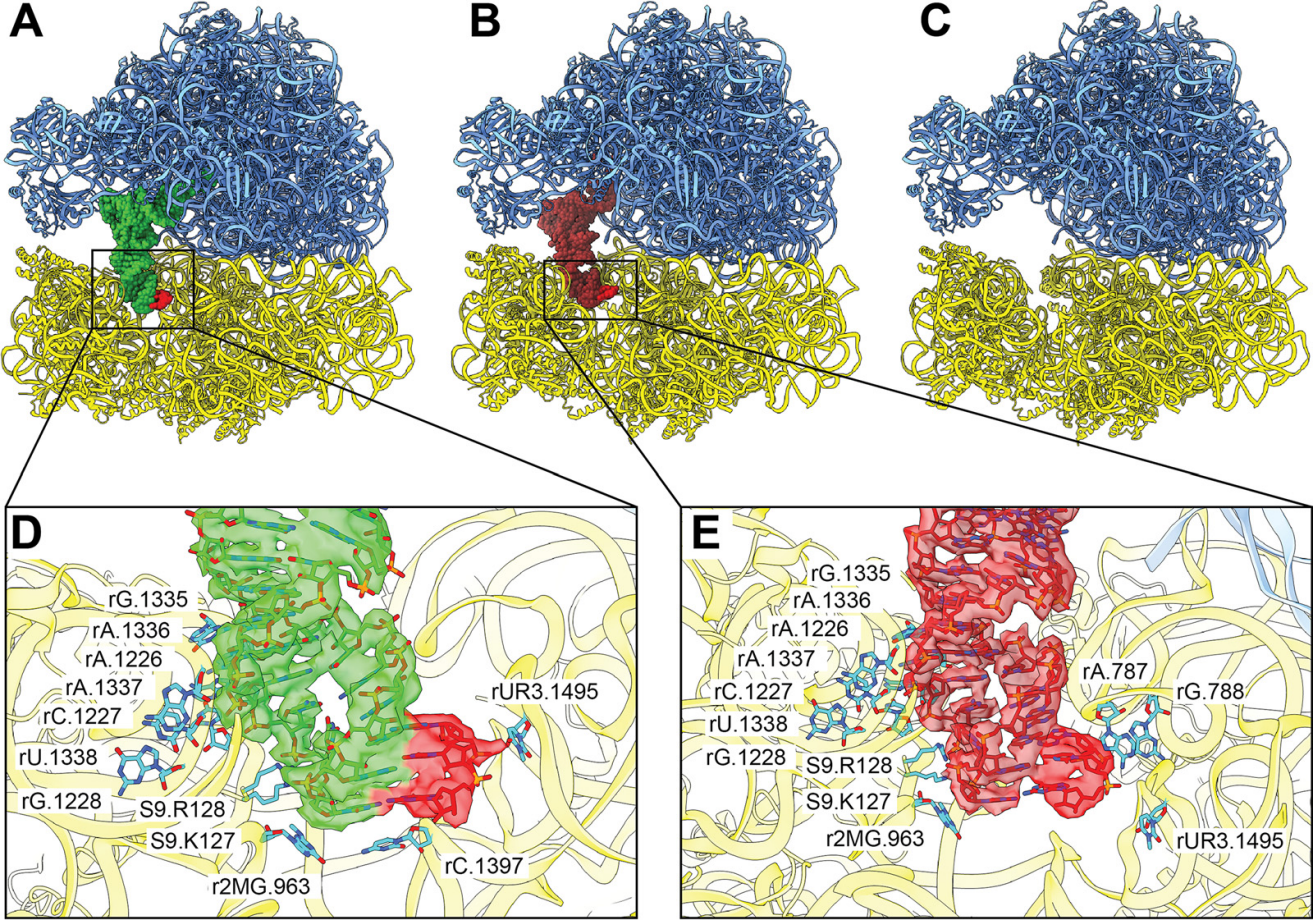
Cryo-EM structures of the A. baumannii ribosome bound by TP-6076. (A to C) Structures of the A. baumannii 70S P-site tRNA, 70S E-site tRNA, and empty 70S ribosomes in complex with TP-6076. The 50S subunit is in blue, 30S is in yellow, P-site tRNA is in green, E-site tRNA is in brown, and mRNA is in red. (D and E) interactions between the P-site and E-site tRNAs and the 30S ribosomal subunits. P-site tRNA, E-site tRNA, and mRNA are shown as green, brown, and red sticks, respectively. Residues within 4 Å are shown as cyan sticks. Cryo-EM densities corresponding to P-site tRNA, E-site tRNA, and mRNA are shown as transparent surfaces and are in green, brown, and red, respectively.

The cryo-EM maps of these three ribosome structures were resolved to resolutions of between 2.38 Å and 3.05 Å (Fig. S4 and Table S3), allowing the unambiguous determination of the 23S rRNA, 5S rRNA, and 28 rProteins of the 50S large ribosomal subunit and the 16S rRNA and 20 ribosomal proteins (20 rProteins) of the 30S small ribosomal subunit (Fig. 2A to C). Using masked classification, a single tRNA molecule was found to occupy either the P site or the E site of the ribosome, giving rise to the P-site tRNA or E-site tRNA 70S cryo-EM structures, respectively. There was also a distinct class of single-particle population where no tRNA was found in the A, P, or E sites, leading to the empty 70S ribosome structure (Fig. 2A to E).

10.1128/mbio.03732-21.4 FIG S4 Final refinements of A. baumannii ribosome–TP-6076 complexes. (A) Representative 2D classes (top left), final locally refined maps (bottom left), and GS-FSC resolutions (right) of the 70S P-site tRNA bound with TP-6076. (B) Representative 2D classes (top left), final locally refined maps (bottom left), and GS-FSC resolutions (right) of the 70S E-site tRNA bound with TP-6076. (C) Representative 2D classes (top left), final locally refined maps (bottom left), and GS-FSC resolutions (right) of empty 70S bound with TP-6076. For all maps, 50S is in blue, while the 30S core is in yellow, and the 30S head is in red. Download FIG S4, JPG file, 2.1 MB. Copyright © 2022 Morgan et al. 2022 Morgan et al. https://creativecommons.org/licenses/by/4.0/ This content is distributed under the terms of the Creative Commons Attribution 4.0 International license.

10.1128/mbio.03732-21.7 TABLE S3 Ribosome cryo-EM data collection and refinement statistics. Download Table S3, DOCX file, 0.02 MB. Copyright © 2022 Morgan et al. 2022 Morgan et al. https://creativecommons.org/licenses/by/4.0/ This content is distributed under the terms of the Creative Commons Attribution 4.0 International license.

**FIG 3:**
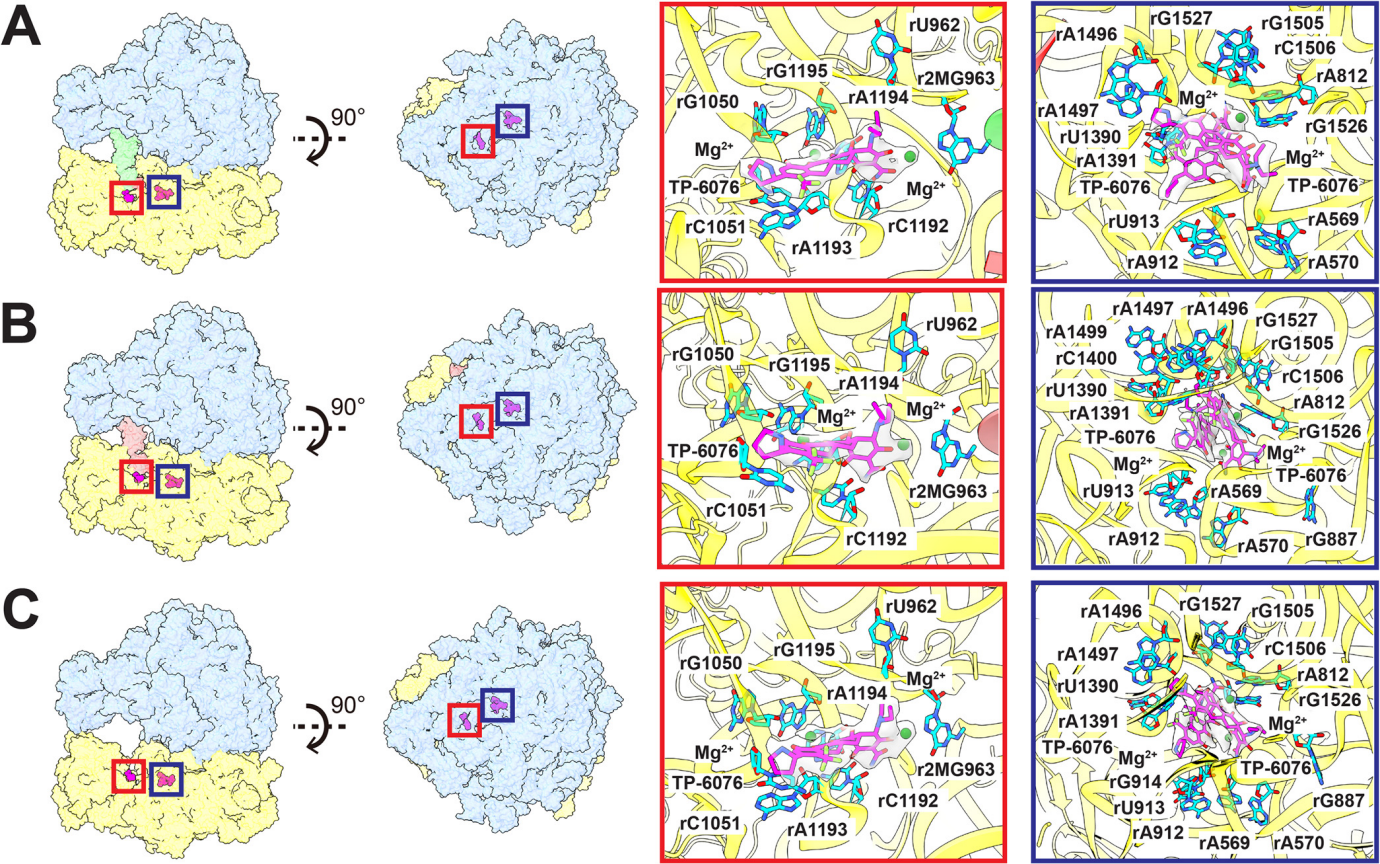
TP-6076 binding sites in the ribosome. Depicted are interactions with TP-6076 in the 70S P-site tRNA, 70S E-site tRNA, and the empty 70S A. baumannii ribosome. Binding sites are conserved independent of the tRNA population. The putative tetracycline binding site in the head of the 30S subunit is highlighted with a red square, while a secondary binding site is highlighted with a blue square. TP-6076 is depicted as magenta sticks, and the corresponding cryo-EM density is shown as a transparent surface. Mg^2+^ is shown as green spheres. Nucleotides within 4 Å of TP-6076 and the coordinated Mg^2+^ ions are highlighted as cyan sticks.

Based on the structural information, there are two binding sites for TP-6076 present in all three 70S ribosome structures. These two TP-6076 binding sites are both located at the 30S small ribosomal subunit (Fig. 3A to C). The first binding site depicts the putative tetracycline binding site, found in the 30S head (Fig. 3A to C). At this site, one TP-6076 molecule occupies this putative binding site stabilized by coordination with two Mg^2+^ ions and a number of electrostatic interactions from nucleotides rU962, r2MG963, rG1050, rC1051, rC1192, rA1193, rA1194, and rG1195. This site overlaps the A-site tRNA pocket of the 30S subunit, posing the possibility that the drug is responsible for blocking the attachment of tRNA to the A site and halting translation elongation, a trademark characteristic of tetracycline antibiotics (6).

The second TP-6076 binding site is located within the core of the small 30S ribosomal subunit (Fig. 3A to C). At this position, two TP-6076 molecules coordinate with two Mg^2+^ ions in the drug binding site. These drug molecules are stabilized by a number of electrostatic interactions via nucleotides rA569, rA570, rA812, rA912, rU913, rU1390, rA1391 rA1496, rA1497, rG1505, rC1506, rG1526, and rG1527. As with the first drug binding site, this site remains consistent throughout the three 70S structures. These two TP-6076 molecules directly interact with helix 44 (h44) of the 16S rRNA, which is vital for translational fidelity and initiation (39). While mutation studies have confirmed the putative tetracycline binding site within the 30S head (40), no systemic studies have been performed to show the importance of the second TP-6076 binding site within the core of the 30S subunit. Whether binding at this site by TP-6076 has biological implications invites further experimentation.

## DISCUSSION

Antibiotic resistance is a major clinical challenge for modern medicine. A. baumannii is a major threat in this regard, where carbapenem resistance is now present in most A. baumannii strains along with an alarming rise in tigecycline and colistin resistance (41, 42). One major reason for this is the overexpression and selective mutation of multidrug efflux pumps (26, 43). Therefore, a greater understanding of this resistance mechanism will aid in the future development of inhibitors that effectively block this efflux process and increase the potency of current antibiotics.

Toward this goal, we have solved the structures of TP-6076 bound to both the A. baumannii multidrug efflux pump AdeJ and the A. baumannii ribosome using cryo-EM. This adds to our recent work in which the structures of AdeJ and the A. baumannii ribosome bound to the tetracycline antibiotic eravacycline (Era) were solved (27, 44, 45). Together, these data highlight the versatility of the multidrug efflux pump AdeJ and its ability to recognize a wide variety of substrates.

**FIG 4:**
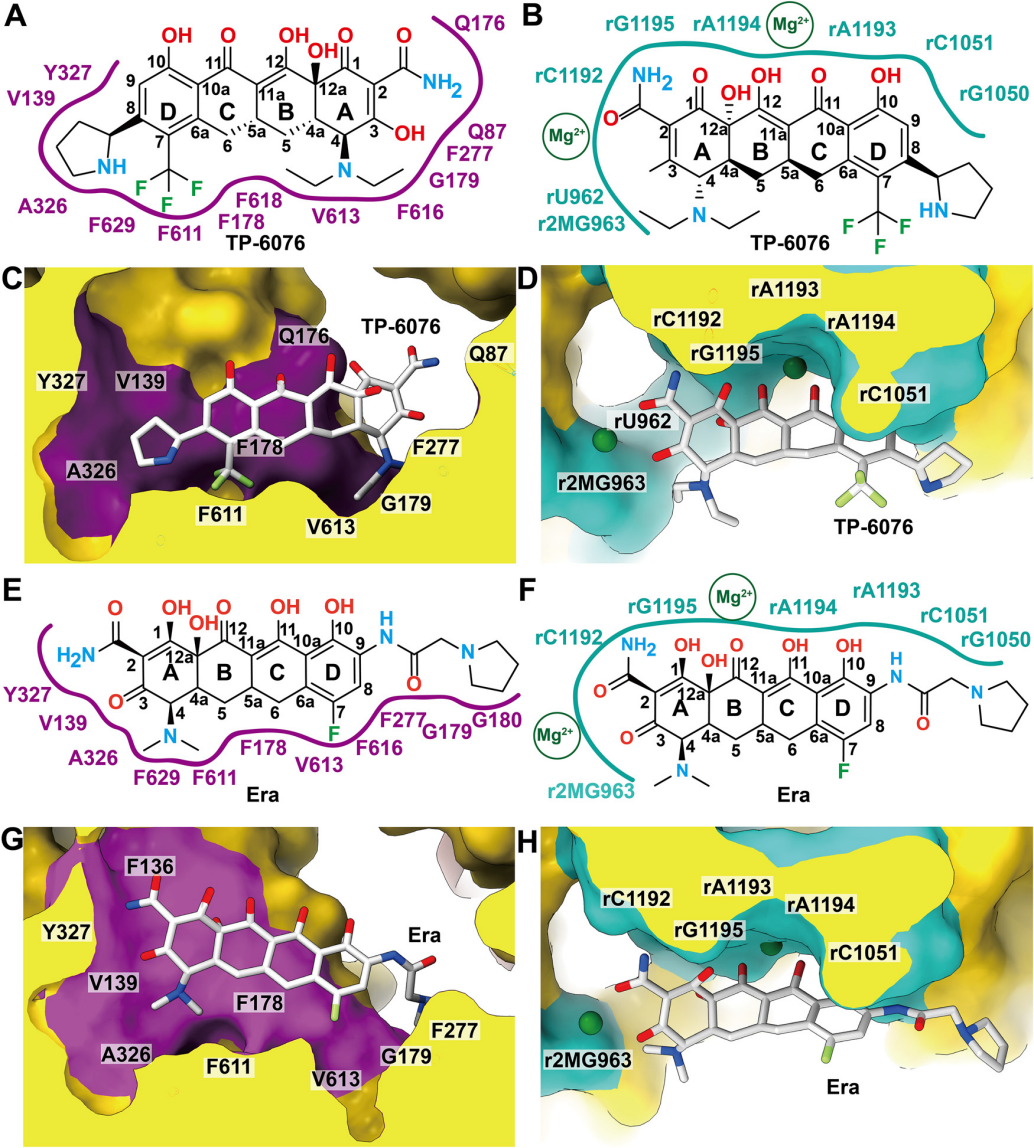
Binding site comparison. (A) 2D diagram of the interaction network of A. baumannii AdeJ and TP-6076. The interaction area is indicated with a magenta line. (B) 2D diagram of the interaction network of the A. baumannii ribosome and TP-6076. The interaction area is shown as a cyan line. Magnesium ions are labeled in a circle with Mg^2+^. (C and D) The TP-6076 binding pocket of AdeJ (C) and the ribosome (D). (E) 2D diagram of the interaction network of AdeJ and Era. The interaction area is indicated with a magenta line. (F) 2D diagram of the interaction network of the ribosome and Era. The interaction area is shown as a cyan line. Magnesium ions are labeled in a circle with Mg^2+^. (G and H) The eravacycline binding pocket of AdeJ (G) and the ribosome (H). The interaction areas of the ligand are shown in magenta and cyan surfaces according to the diagrams in panels A and B, respectively. The binding pocket of AdeJ is more sterically restrictive than the binding pocket in 30S of the ribosome.

TP-6076 binding to AdeJ is accomplished mainly through hydrophobic interactions, with additional stabilization via two unique electrostatic interactions. To accomplish this, the hydrophobic face of the TP-6076 molecule containing positions 4 to 7 is utilized (Fig. 4A and C). All hydrophobic residues within the distal binding pocket of AdeJ (V139, F178, G179, F277, A326, Y327, F611, V613, F616, and F629) participate in both Era and TP-6076 binding. Surprisingly, in order to better accommodate the aminoethyl and trifluoromethyl ring modifications and the shorter pyrrolidine group, TP-6076 is flipped relative to Era in AdeJ (Fig. 4A, C, E, and G). This flipped pose then allows Q87 and Q176 to form additional electrostatic interactions with the carboxamide group at position 2 of ring A. This flipped pose, while still utilizing the same hydrophobic face of the tetracycline moiety, highlights the versatility of the AdeJ pump in substrate recognition and extrusion.

In the ribosome, TP-6076 occupies a site nearly identical to that of Era in the putative tetracycline binding site in the head of the 30S ribosomal subunit (Fig. 3A to C and Fig. 4B, D, F, and H). In both structures, two Mg^2+^ ions coordinate with the tetracycline core, and binding is stabilized through favorable electrostatic interactions with residues r2MG963, rG1050, rC1051, rC1192, rA1193, rA1194, and rG1195. This shows that despite modifications to the drug, TP-6076 likely maintains tetracycline antimicrobial properties, in agreement with previous biological studies (4, 5).

Unlike Era, where a second ligand binding site is found in the 50S large ribosomal subunit, a second TP-6076 site is found in the core of the 30S small ribosomal subunit. In this site, two TP-6076 molecules coordinate with two Mg^2+^ ions and are stabilized by a number of electrostatic interactions. The TP-6076 residues at this site interact directly with h44 of the 16S rRNA. This helix is responsible for maintaining coding fidelity and is the main target of aminoglycoside antibiotics such as kanamycin and gentamicin (10, 11, 39). Therefore, this site may play a role in the overall efficacy of TP-6076 and invites further experimentation.

It is known that the binding affinities of drugs for multidrug efflux pumps are in the micromolar range (28, 34, 37, 46–50). Although the strengths of antibiotic binding within the 70S ribosomes are not as commonly studied, there is evidence that these binding affinities can be substantially strong in comparison. For example, the ribosomes bind macrolides with dissociation constants in the nanomolar range (51, 52). However, the ribosomes also bind several drugs, including tetracyclines (53) and aminoglycosides (54), within the micromolar range, where these binding affinities are similar to those for multidrug efflux pumps.

From our cryo-EM data, it is clear that AdeJ utilizes the hydrophobic properties of tetracyclines for recognition, while its electrostatic properties are vital for interactions with the ribosome. This conserved mode of recognition is evident when comparing the binding of both TP-6076 and Era to AdeJ (Fig. 4). Interestingly, in order to accommodate modifications to the hydrophobic face of TP-6076, the ligand is present in a flipped orientation relative to Era (Fig. 4A, C, E, and G) in AdeJ, while the ribosome binding site is nearly identical (Fig. 4B, D, F, and H). As the tetracycline class of antibiotics utilizes electrostatic interactions to bind and occupy the same respective putative tetracycline site in the 70S ribosomes (11), and both TP-6076 and Era employ hydrophobic interactions to specifically contact the AdeJ pump, we believe that this trend would remain consistent with most tetracycline drugs. Our experimental result underscores the phenomenon that the predominant interaction between tetracyclines and 70S ribosomes is electrostatic in nature, whereas the prevalent association between tetracyclines and multidrug efflux pumps is hydrophobic in character. This notable difference in substrate recognition between 70S and AdeJ may be exploited in future tetracycline-based drugs, where modifications to only the hydrophobic side of the tetracycline core will significantly hinder recognition by multidrug efflux pumps like AdeJ but maintain binding to the ribosome and, therefore, its antimicrobial properties.

## MATERIALS AND METHODS

### Expression and purification of A. baumannii AdeJ.

AdeJ from A. baumannii AB0057 was expressed and purified as previously described (27). In short, AdeJ was cloned into a pET15b vector with a 6×His tag at the N terminus and confirmed by DNA sequencing. The plasmid was transfected into E. coli BL21(DE3) Δ*acrB* cells and grown in 6 L of lysogeny broth (LB) medium with 100 μg/mL of ampicillin at 37°C. At an optical density at 600 nm (OD_600_) of 0.5, the expression of AdeJ was induced with 0.2 mM isopropyl-β-d-thiogalactopyranoside (IPTG). Bacteria were harvested after 4 h of induction. Cells were resuspended in low-salt buffer (100 mM sodium phosphate [pH 7.4], 10% glycerol, 5 mM EDTA, and 1 mM phenylmethanesulfonyl fluoride [PMSF]) and disrupted with a French pressure cell. The collected membrane fraction was washed twice with high-salt buffer (20 mM sodium phosphate [pH 7.4], 2 M KCl, 10% glycerol, 5 mM EDTA, and 1 mM PMSF) and once with final buffer (20 mM Na-HEPES [pH 7.5] and 1 mM PMSF). Membrane protein was solubilized using 2% (wt/vol) *n*-dodecyl-β-d-maltoside (DDM) for 3 h. Insoluble material was removed by centrifugation at 100,000 × *g*, and the extracted protein was purified using a Ni-nitrilotriacetic acid (NTA) column. The purity of AdeJ (>95%) was determined via SDS-PAGE gels stained with Coomassie brilliant blue. The purified protein was then dialyzed against 20 mM Na-HEPES (pH 7.5) and concentrated to 7 mg/mL (60 μM) in a solution containing 20 mM Na-HEPES (pH 7.5) and 0.05% DDM.

### AdeJ-nanodisc preparation.

AdeJ was assembled into nanodiscs using a mixture of 20 μM AdeJ, 45 μM membrane scaffold protein (MSP) (1E3D1), and 930 μM E. coli total lipid extract. The mixture was incubated at room temperature for 15 min. To remove the detergent, 0.8 mg/mL prewashed Bio-Beads (Bio-Rad) was added. The mixture was incubated on ice for 1 h, followed by incubation at 4°C overnight. The sample was filtered using a 0.22-μm nitrocellulose filter to remove Bio-Beads and purified from empty nanodiscs using a Superose 6 column (GE Healthcare) in a solution containing 20 mM Tris-HCl (pH 7.5) and 100 mM NaCl. Fractions containing the trimeric AdeJ-nanodisc complex were collected for cryo-EM sample preparation.

### Purification of the A. baumannii ribosome.

Ribosomes were purified directly from A. baumannii AB0057 as previously described (55). In brief, A. baumannii cells were lysed in a solution containing 20 mM Tris (pH 7.5), 50 mM magnesium acetate (MgOAc), 100 mM NH_4_Cl, 1 mM dithiothreitol (DTT), and 0.5 mM EDTA (56) using a French pressure cell. The lysate was centrifuged at 30,000 × *g* for 30 min to pellet insoluble material. Ribosomes were pelleted from the supernatant using a sucrose cushion buffer (lysis buffer with 1.1 M sucrose) and centrifuged for 16 h at 100,000 × *g*. Ribosome-containing pellets were stored at −80°C until further use.

Sucrose gradient purification using a sucrose gradient buffer (20 mM HEPES, 14 mM MgOAc, 100 mM KCl, 0.2 mM DTT, and 0.1 mM PMSF with 10% and 40% sucrose and mixed using a gradient maker) and 16 h of centrifugation at 100,000 × *g* was used to increase 70S purity. Fractions containing 70S particles were collected, concentrated, exchanged into ribosome buffer (20 mM HEPES-KOH [pH 7.5], 10 mM MgOAc, and 100 mM KCl), aliquoted, flash-frozen in liquid nitrogen, and stored at −80°C until further use.

### Cryo-EM sample preparation.

A total of 10 μM AdeJ-nanodisc was incubated with 20 μM TP-6076 for 1 h prior to grid preparation to create the AdeJ–TP-6076 complex. The sample was applied to glow-discharged holey carbon grids (Quantifoil Cu R1.2/1.3, 300 mesh), blotted for 5 s, and then plunge-frozen in liquid ethane using a Vitrobot (Thermo Fisher). For the A. baumannii ribosome, 100 nM ribosome sample was incubated with 50 μM TP-6076 for 2 h prior to grid preparation to create the ribosome–TP-6076 complex. Grids were plunge-frozen with a Vitrobot using a 15-s blot time and graphene oxide-coated Quantifoil R1.2/1.3 grids. All grids were transferred to cartridges prior to data collection.

### Data collection.

For the AdeJ–TP-6076 complex, data were collected on a Titan Krios system equipped with a K3 direct electron detector (Gatan). Data were collected in superresolution mode at a magnification of ×81,000, resulting in a physical pixel size of 1.08 Å/pix (superresolution of 0.54 Å/pix). Each micrograph was collected over 40 frames with a total dose of 36 e^−^/Å^2^ over 2 s using SerialEM (57). For the ribosome–TP-6076 complex, data were collected on a Titan Krios system equipped with a Falcon III detector (Gatan). Data were collected in linear mode at a magnification of ×75,000, corresponding to a physical pixel size of 1.089 Å/pix. Each micrograph was collected over 60 frames over 1.9 s with a total dose of 50 e^−^/Å^2^ using EPU (Thermo Fisher Scientific).

### Data processing.

For AdeJ, superresolution stacks were aligned and binned by 2 using patch motion, and contrast transfer function (CTF) was estimated using patch CTF in cryoSPARC v3 (58). Blob picker followed by two-dimensional (2D) classification was used to generate templates for template picker (see Fig. S1 in the supplemental material), which picked an initial pool of 2,975,155 particles. 2D classifications followed by *ab initio* and heterogeneous three-dimensional (3D) classifications were used to remove false picks and contamination from ice and carbon. 3D classification using 3D variability analysis on the remaining 595,961 particles resulted in a single high-resolution class for refinement. Nonuniform refinement followed by masked local refinement with nonuniform sampling resulted in 2.91-Å-resolution maps of the AdeJ–TP-6076 complex based on the gold-standard Fourier shell correlation (GS-FSC) (Fig. S1 and Table S1). The final maps were modified using resolve cryo-EM (59) from the phenix suite of programs (60).

For the ribosome, image stacks were motion corrected using patch motion, and patchCTF was used to estimate CTF (58). Blob picker using a subset of micrographs was used to generate templates for template picker, resulting in an initial particle stack of 725,635 particles (Fig. S3). 2D classifications followed by *ab initio* and heterogeneous 3D classifications were used to generate initial templates. Using a modified “build-and-retrieve” approach (61), initial templates were used to retrieve particles from the initial particle stack, resulting in markedly higher final particle counts. 70S classes were separated based on tRNA populations in the P and E sites using 3D variability analysis in cryoSPARC (58) and refined using homogeneous refinement. The final refinement of each 70S class was performed by using masked local refinement with nonuniform sampling and dividing each map into three sections: 50S, 30S core, and 30S head (Fig. S4). Final maps were sharpened using resolve cryo-EM in phenix (59). Locally refined map segments were used to build the final model for each class (70S P-site tRNA, 70S E-site tRNA, and empty 70S), and the final composite map for visualization was created using vop maximum in chimera (62).

### Model building and refinement.

Models for the TP-6076–AdeJ complex and the ribosome (70S P-site tRNA, 70S E-site tRNA, and empty 70S) were based on the cryo-EM maps. Previously determined structures for AdeJ (27) and the A. baumannii ribosome (55) were used as starting models. Starting models were aligned to the cryo-EM maps using chimera (62). The width of the extrusion tunnel along with the periplasmic cleft state and PMF measurements were used to classify protomer states (Fig. S2 and Table S2). For the ribosome, Mg^2+^ ions directly coordinated with each TP-6076 molecule were manually added in Coot (63). Models for all structures were built using Coot (63) and refined using phenix.real_space_refine (64) from the phenix suite (60). Final structures were evaluated using MolProbity (65), with final refinement statistics included in Tables S1 and S3.

### Data availability.

Atomic coordinates and the EM map for the AdeJ–TP-6076 complex have been deposited in the RCSB Protein Data Bank (PDB) under accession number 7RY3 and the Electron Microscopy Data Bank (EMDB) under accession number EMD-24732. Atomic coordinates for TP-6076 in complex with A. baumannii P-site tRNA 70S, E-site tRNA 70S, and empty 70S have been deposited in the PDB under accession numbers 7RYF, 7RYG, and 7RYH and in the EMDB under accession numbers EMD-24738, EMD-24739, and EMD-24740, respectively.
