# Supplementary figures and images for: An Analysis of the Novel Fluorocycline TP-6076 Bound to Both the Ribosome and Multidrug Efflux Pump AdeJ from Acinetobacter baumannii
Source: mBio. 2022 Feb 1;13(1):e03732-21. doi: 10.1128/mbio.03732-21 (PMC8805024; doi:10.1128/mbio.03732-21)

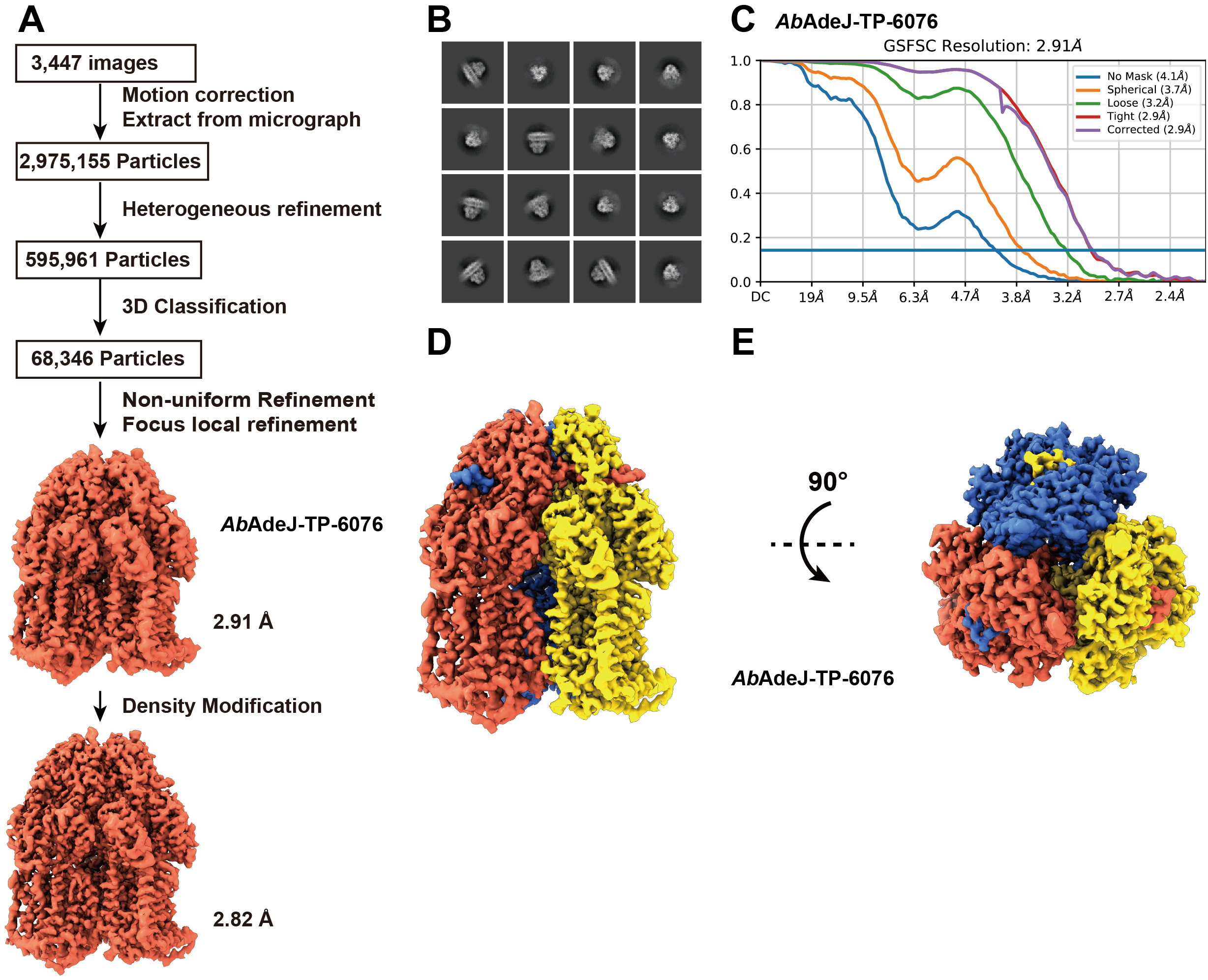

Supplement: FIG S1 [file mbio.03732-21-sf001.jpg]

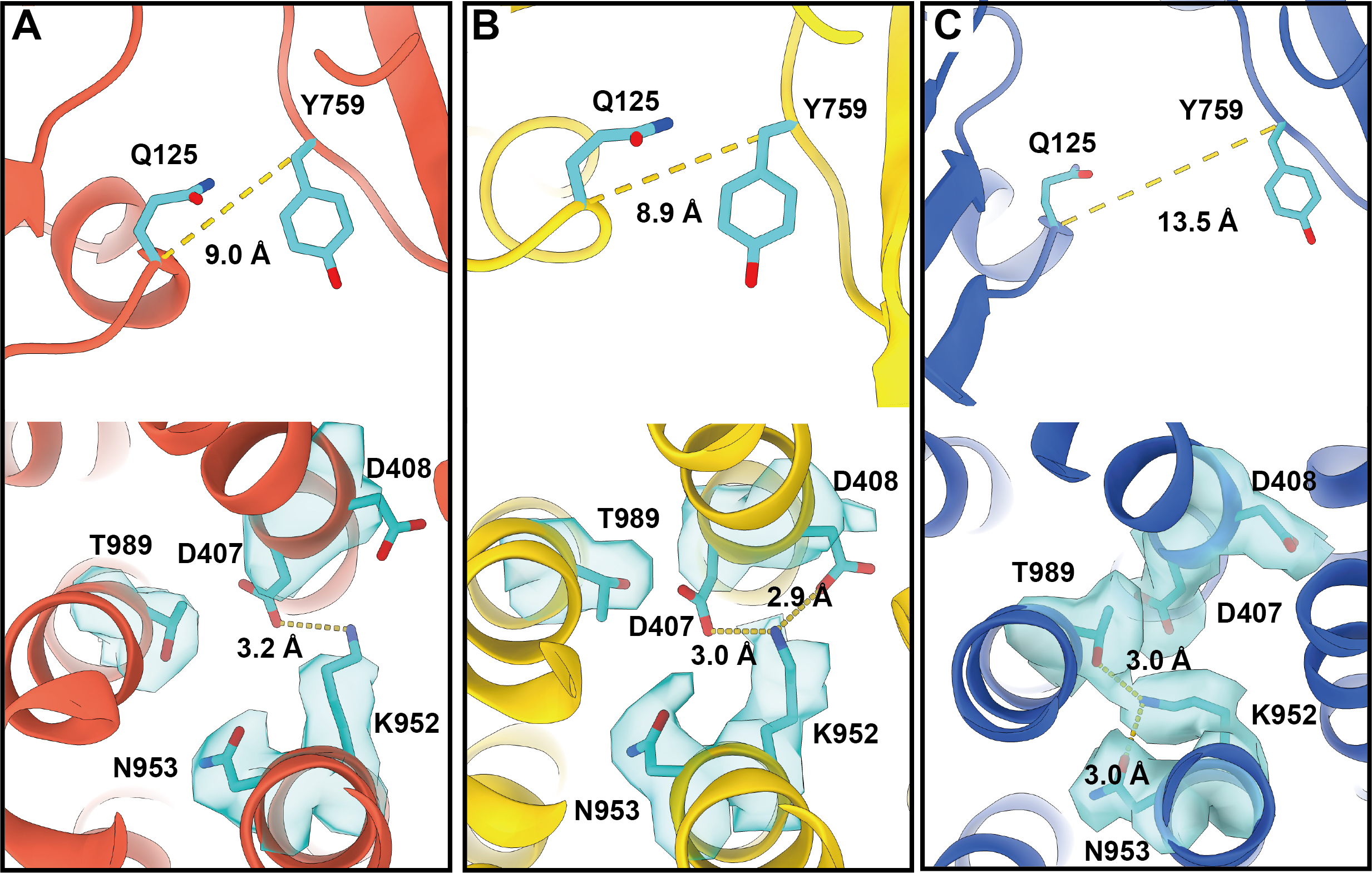

Supplement: FIG S2 [file mbio.03732-21-sf002.jpg]

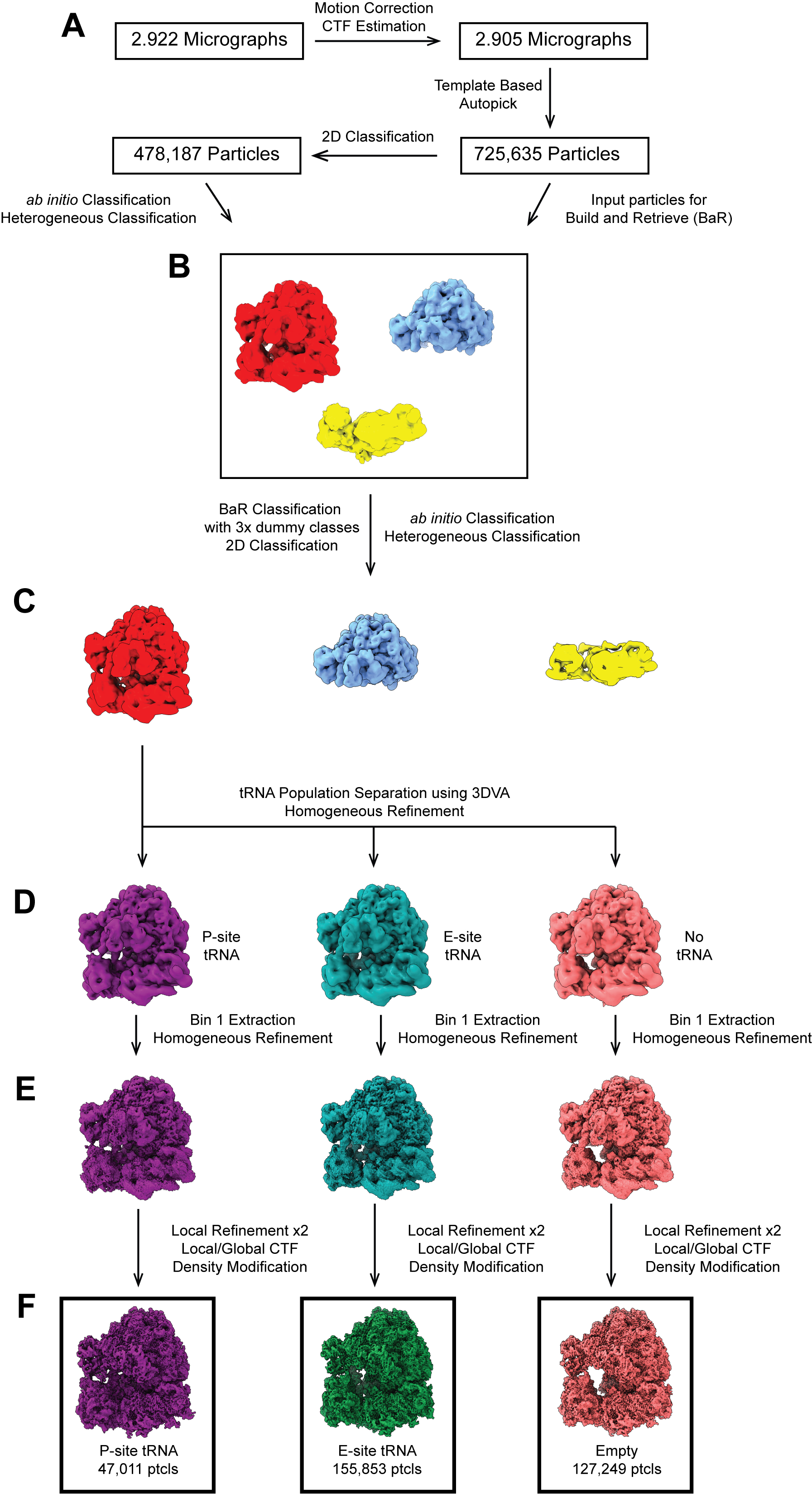

Supplement: FIG S3 [file mbio.03732-21-sf003.jpg]

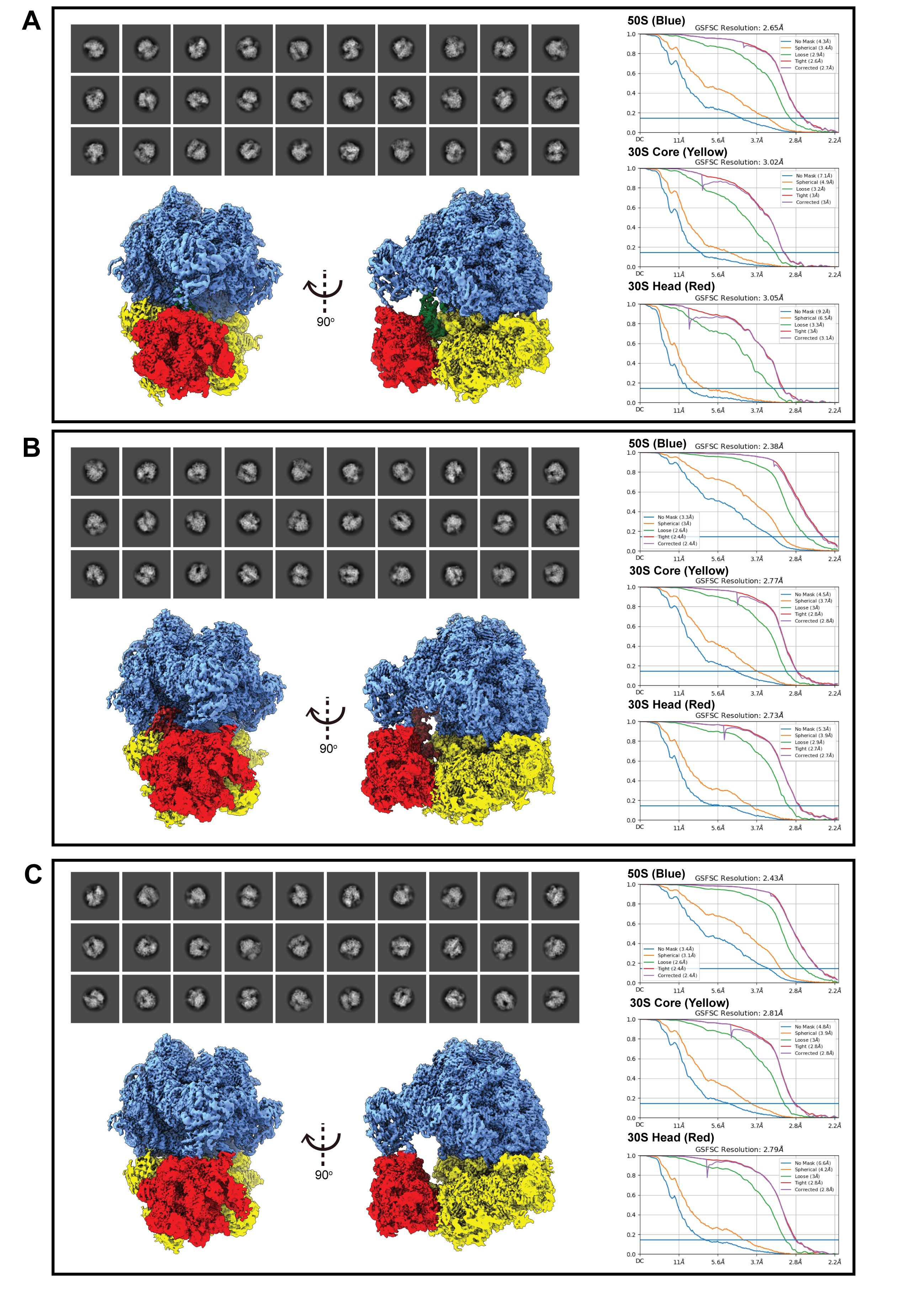

Supplement: FIG S4 [file mbio.03732-21-sf004.jpg]
