# Supplementary material for: An Analysis of the Novel Fluorocycline TP-6076 Bound to Both the Ribosome and Multidrug Efflux Pump AdeJ from Acinetobacter baumannii
Source: mBio. 2022 Feb 1;13(1):e03732-21. doi: 10.1128/mbio.03732-21 (PMC8805024; doi:10.1128/mbio.03732-21)
Supplement: TABLE S1 [file mbio.03732-21-st001.docx]

| **Table S1. AdeJ cryo-EM data collection and refinement statistics.** | | | | | | | |
| --- | --- | --- | --- | --- | --- | --- | --- |
| **Data collection** |  |  |  |  |  |  | |
| Magnification | 81,000 | | | | | | |
| Voltage (kV) | 300 | | | | | | |
| Electron Microscope | Krios-GIF-K3 | | | | | | |
| Defocus (um) | -1.0 to -2.5 | | | | | | |
| Total exposure time (s) | 2 | | | | | | |
| Energy filter width (eV) | 20 | | | | | | |
| Pixel size (Å) | 1.08 (0.54) | | | | | | |
| Total dose (e^-^/ Å^2^) | 36 | | | | | | |
| Number of frames | 40 | | | | | | |
| Number of micrographs | 3,447 | | | | | | |
| Initial particle images (no.) | 2,975,155 | | | | | | |
| **Refinement** | **AdeJ-TP-6076** | | | | | |  |
| Total Particles (no.) | 68,346 | | | | | |  |
| Symmetry | C1 | | | | | |  |
| GS-FSC Resolution (0.143, Å)^a^ | 2.91 | | | | | |  |
| Model composition |  | | | | | |  |
| Protein residues | 3,140 | | | | | |  |
| Ligands | 1 | | | | | |  |
| r.m.s.d. |  | | | | | |  |
| Bond lengths (Å) | 0.011 | | | | | |  |
| Bond angles (°) | 1.547 | | | | | |  |
| **Validation** | **AdeJ-TP-6076** | | | | | |  |
| MolProbity score | 1.68 | | | | | |  |
| Clash score | 5.51 | | | | | |  |
| Ramachandran plot |  | | | | | |  |
| Favored (%) | 98.37 | | | | | |  |
| Allowed (%) | 1.60 | | | | | |  |
| Disallowed (%) | 0.03 | | | | | |  |
| CC Mask | 0.73 | | | | | |  |
