# Supplementary material for: An Analysis of the Novel Fluorocycline TP-6076 Bound to Both the Ribosome and Multidrug Efflux Pump AdeJ from Acinetobacter baumannii
Source: mBio. 2022 Feb 1;13(1):e03732-21. doi: 10.1128/mbio.03732-21 (PMC8805024; doi:10.1128/mbio.03732-21)
Supplement: TABLE S2 [file mbio.03732-21-st002.docx]

**Table S2. Classification of AdeJ protomer states.**

| **Protomer** | **Cleft State** | **Exit site distance, Q125 to Y759** | **Hydrogen-bonded distance, K952 to** | | | | **Protomer Assignment** |
| --- | --- | --- | --- | --- | --- | --- | --- |
|  |  |  | **D407 (Å)** | **D408 (Å)** | **N953 (Å)** | **T989 (Å)** |  |
| AdeJ-TP-6076, A | Closed | 13.5 | - | - | 3.0 | 3.0 | Extrusion |
| AdeJ-TP-6076, B | Open | 9.0 | 3.2 | - | - | - | Access |
| AdeJ-TP-6076, C | Open | 8.9 | 3.0 | 2.9 | - | - | Binding |

AdeJ protomers were defined using three criteria; state of the periplasmic cleft (open or closed), measurement of the exit site (distance between Cα atoms of Q125 and Y759) and hydrogen bond distances of the PTC (K952 to D407, D408, N953 and T989). Using these results, protomers were assigned as either resting, access, binding and extrusion.
