# Supplementary material for: An Analysis of the Novel Fluorocycline TP-6076 Bound to Both the Ribosome and Multidrug Efflux Pump AdeJ from Acinetobacter baumannii
Source: mBio. 2022 Feb 1;13(1):e03732-21. doi: 10.1128/mbio.03732-21 (PMC8805024; doi:10.1128/mbio.03732-21)
Supplement: TABLE S3 [file mbio.03732-21-st003.docx]

| **Table S3. Ribosome cryo-EM data collection and refinement statistics.** | | | | | | | |
| --- | --- | --- | --- | --- | --- | --- | --- |
| **Data collection** |  |  |  | |  |  |  |
| Magnification | 75,000 | | | | | | |
| Voltage (kV) | 300 | | | | | | |
| Electron Microscope | Krios-Falcon III | | | | | | |
| Defocus (um) | -0.8 to -2.2 | | | | | | |
| Total exposure time (s) | 1.9 | | | | | | |
| Pixel size (Å) | 1.089 | | | | | | |
| Total dose (e^-^/ Å^2^) | 50 | | | | | | |
| Number of frames | 60 | | | | | | |
| Number of micrographs | 2,922 | | | | | | |
| Initial particle images (no.) | 725,635 | | | | | | |
| Final particle images (no.) | 478,187 | | | | | | |
| **Refinement** | **70S P-site-tRNA** | **70S E-site-tRNA** | | **70S Empty** | | | |
| Total Particles (no.) | 47,011 | 155,853 | | 127,249 | | | |
| GS-FSC Resolution (0.143, Å)^a^ |  |  | |  | | | |
| 50S | 2.65 | 2.38 | | 2.43 | | | |
| 30S Core | 3.02 | 2.77 | | 2.81 | | | |
| 30S Head | 3.05 | 2.73 | | 2.79 | | | |
| Model composition |  |  | |  | | | |
| Chains | 53 | 53 | | 51 | | | |
| Protein residues | 5,497 | 5,505 | | 5,499 | | | |
| Nucleotides | 4,408 | 4,408 | | 4,328 | | | |
| Mg^2+^ | 4 | 4 | | 4 | | | |
| TP-6076 | 3 | 3 | | 3 | | | |
| r.m.s.d. |  |  | |  | | | |
| Bond lengths (Å) | 0.002 | 0.002 | | 0.002 | | | |
| Bond angles (°) | 0.535 | 0.539 | | 0.544 | | | |
| **Validation** | **70S P-site-tRNA** | **70S E-site-tRNA** | | **70S Empty** | | | |
| MolProbity score | 1.32 | 1.58 | | 1.54 | | | |
| Clash score | 4.25 | 3.68 | | 3.73 | | | |
| Ramachandran plot |  |  | |  | | | |
| Favored (%) | 97.43 | 97.24 | | 97.67 | | | |
| Allowed (%) | 2.50 | 2.64 | | 2.22 | | | |
| Disallowed (%) | 0.07 | 0.11 | | 0.11 | | | |
| CC Mask | 0.72 | 0.73 | | 0.72 | | | |

^a^Gold-Standard Fourier Shell Correlation; refinements split into three regions for each structure.
